# Multidomain intervention for preventiing cognitive decline among older adults with mild cognitive impairment ‐ Interim results from TINAGE trial

**DOI:** 10.1002/alz70861_108294

**Published:** 2025-12-23

**Authors:** Saritha Susan Vargese, Pramod Thomas, Thomas Mathew, Deepak Varughese Thomas

**Affiliations:** ^1^ Believers Church medical College Hospital, Tiruvalla, Kerala India

## Abstract

**Background:**

Existing pharmacological treatments have shown only modest benefits, underscoring the need for alternative approaches such as multidomain interventions for preventing cognitive decline among at risk individuals.

**Method:**

A 2 year interventional study is ongoing to prevent cognitive decline using a multidomain intervention package among older adults between 60‐75 years with mild cognitive impairment. Multidomain package includes nutritional advice, physical activity, cognitive stimulation therapy, management of vascular risk factors and social engagement sessions.

**Result:**

During the one‐year follow‐up, the intervention group showed a significant increase in mean cognitive scores (Addenbrooke’s cognitive examination), rising from 73.94 to 82.32 (p < 0.001). The control group also demonstrated a modest but statistically significant improvement, with scores increasing from 72.28 to 74.21 (p < 0.001). Notably, there was a significant difference between the intervention and control groups at the end of the one‐year follow‐up.

**Conclusion:**

The interim results are promising and if found to be effective could be integrated to primary health care in similar settings.

